# Altered monocytic phenotypes are linked with systemic inflammation and may be linked to mortality in dialysis patients

**DOI:** 10.1038/s41598-019-55592-y

**Published:** 2019-12-13

**Authors:** Sabine Brandt, Lara Ewert, Florian G. Scurt, Charlotte Reichardt, Jonathan A. Lindquist, Xenia Gorny, Berend Isermann, Peter R. Mertens

**Affiliations:** 1 Department of Nephrology and Hypertension, Diabetes and Endocrinology, Otto-von-Guericke University Magdeburg, Magdeburg, Germany; 2 Institute of Clinical Chemistry and Pathobiochemistry, Otto-von-Guericke University Magdeburg, Magdeburg, Germany

**Keywords:** Sepsis, Haemodialysis

## Abstract

The major causes for increased morbidity and mortality among chronic kidney disease patients are cardiovascular diseases and infection. A causal link between an activated immune system and aggravated atherosclerosis has been postulated that skews the system towards inflammatory responses. Previously, we demonstrated a positive association of pro-inflammatory cytokines with monocytic Y-box binding protein-1 (YB-1) expression and vessel wall infiltration in hemodialysis patients. Here, we question whether the responsiveness and cytokine repertoire of monocytes is altered by pre-activation and how this correlates with survival. EDTA whole blood from hemodialysis patients (n = 45) and healthy controls (n = 34) was collected and leukocytes challenged with LPS. The distribution of monocyte subsets, YB-1_acetyl_ content, and serum cytokine levels were determined. Compared to controls, dialysis patients have fewer classical (Mo1) and more intermediate (Mo2) and non-classical (Mo3) monocytes. In response to LPS, the Mo2 subset significantly increases (p < 0.001) in control subjects, but not in hemodialysis patients; increased CD86 expression indicates a positive response to LPS. Based on the changes within Mo2, subjects could be classified as responders or non-responders: 60% non-responders were seen in the dialysis cohort *versus* only 35% among healthy controls. YB-1 acetylation is higher in dialysis patients, independent of LPS stimulation. In this small cohort with 72 months follow-up period intracellular YB-1_acetyl_ levels, IL-6, uPAR, and IP10 correlated with excess mortality in the dialysis cohort. Changes in YB-1 acetylation and serum cytokines may, at a given time point, possibly predict the long-term outcome and thus provide a legacy effect in hemodialysis patients.

## Introduction

Infection is the second leading cause of death in patients with end stage renal disease (ESRD), following cardiovascular disease^1,2^. Lipopolysaccharide (LPS) is the most potent and well characterized bacterial endotoxin, which binds to CD14 and TLR4 on monocytes, macrophages, and neutrophils, thereby activating signaling pathways leading to NF-ĸB activation. LPS triggers a variety of cellular responses, including cytokine production, arachidonic acid metabolite release, and the generation of reactive oxygen and nitrogen species to promote pathophysiological reactions^3,4^.

In healthy human blood, monocytes comprise 5 to 10% of the circulating leukocytes. Monocytes can be subdivided according to the surface expression of CD14 and CD16 into “classical” CD14^++^CD16^−^ monocytes (Mo1), representing ~90% of all monocytes. The remaining 10% are CD16 positive; the majority being CD14^+^CD16^++^ “non-classical” (Mo3) with a minor population of “intermediate” CD14^++^CD16^+^ (Mo2) monocytes. CD16 surface expression is considered a marker of monocyte maturation, since “non-classical” Mo3 monocytes are most similar to tissue macrophages with respect to their surface marker expression^5^. In dialysis patients, a shift towards pre-activated monocytic subpopulations (Mo2 and Mo3) has been reported^6^. Mo2 and Mo3 are a primary source of pro-inflammatory cytokines that contribute to the inflammatory milieu^7^.

Recently, we showed that the acetylation status of Y-box binding protein-1 (YB-1; lysines 301/304) is enhanced in circulating monocytes of dialysis patients compare to healthy controls^8^. YB-1 is a ubiquitously expressed member of the cold shock protein family, which regulates transcription, RNA splicing, and translation via its nucleic acid binding activities. Multiple genes with chemoattractive activities are targets of YB-1; e.g. the chemokine (C-C motif) ligand 5 (CCL5)/RANTES, CCL2/MCP1, KC/CXL1, M-CSF, IL-10, and IL-17^9–14^. Post-translational modification of YB-1 dictates its subcellular localization^15–17^, thereby influencing its activities^12,13,18^.

Although it is known that monocytes from dialysis patients display a “pre-activated” phenotype^19^, studies analyzing the relationship of the “pre-activation” status in monocytes with LPS responsiveness and mortality are missing. We hypothesized that monocyte exhaustion due to pre-activation may be linked to enhanced mortality of dialysis patients. To test this hypothesis, we defined the monocyte phenotypes by their surface marker expression profile and compared the LPS responsiveness of monocytes from dialysis patients *versus* healthy controls. Furthermore a set of inflammatory marker proteins was selected that is known to perform short and long-range effects in inflammation and also likely altered due to monocyte function^8,20,21^. Our second hypothesis was that monocyte phenotypic changes correlate with altered circulating cytokine levels. Finally, given that there was a long follow up period lasting 6 years, our quest was to test for potential legacy effects on overall survival. Such legacy effects are already described for probiotics on the gut microbiota^22^. For dialysis patients, such legacy effects have not been previously analyzed. Since 15% of the deaths among patients with ESRD are attributable to infectious causes, such effects must be strongly considered, especially because they may be amenable to direct therapy^23^.

## Materials and Methods

### Control and dialysis cohorts

The study was approved by the local ethics committee at the University Hospital Magdeburg (EK 73/90) and all experiments were performed in accordance with relevant guidelines and regulations. 45 patients who underwent chronic hemodialysis thrice weekly at the KfH Magdeburg were enrolled following informed written consent. Clinical data were retrieved from the medical records and by interviews. Diabetes mellitus was diagnosed according to the German Diabetes Society guidelines^24^. The dialysis cohort included 31 males and 14 females with an average age of 63 ± 16 years. Patients were on regular hemodialysis treatment since 4.1 ± 4 years (range: 0.2–22 years). Blood from 34 healthy volunteers (49 years on average; range: 40–62 years; 21 males and 13 females) recruited from the Institute of Transfusion Medicine, Otto-von-Guericke University, Magdeburg served as control cohort. Venous blood (10 ml) was collected in EDTA-containing vials from each patient before the start of a dialysis session.

### LPS stimulation

Whole blood (200 µl) was mixed 1:1 with complete medium (RPMI, 10% FCS), supplemented with either lipopolysaccharide (LPS, Sigma L-2654 in PBS, final concentration 5 ng/ml) or PBS alone and incubated for 2 h at 37 °C.

### Cytokine determination

Cytokine quantification was performed as described^8^. All analytes were measured by magnetic luminex screening assay using Human Premixed Multi-Analyte Kit (R&D Systems) according to the manufacturers’ instructions. Measurements were performed with a Bioplex200 Analyzer equipped with Bio-Plex Manager^TM^ Software (Bio-Rad).

### Antibody staining and flow cytometry

Following stimulation, the blood-medium mixture was diluted with 2 ml FACS buffer (PBS supplemented with 5% FCS, 0.5% BSA, 0.07% NaN_3_), gently mixed, and centrifuged for 5 minutes at 1,300 rpm at room temperature. Erythrocytes were lysed by resuspending the cell pellet in 2 ml lysis buffer (BD Pharm Lyse™) followed by incubation for 10 min at room temperature, followed by two additional washing steps with FACS buffer. For intracellular staining, cells were permeabilized by addition of 1 ml 50% methanol and washed twice with FACS buffer. Primary antibodies were added at the indicated dilution, incubated for 30 min at R/T, washed twice, and incubated for 30 min at R/T in darkness in a mixture containing surface as well as secondary FITC-labeled antibodies. After two washes with FACS buffer cells were fixed in 200 μl 4% paraformaldehyde in PBS (pH 7.4) for 15 min. The pellet was resuspended in 200 µl PBS and analyzed by flow cytometry. Surface marker antibodies: CD86-PE, clone HA5.2B7 (1:20), Beckman-Coulter, Krefeld, Germany; CD16-APC, clone 3G8 (1:20), Invitrogen, Karlsruhe, Germany; and CD14-PerCP, clone Mϕ9 (1:20), BD Biosciences. Intracellular marker antibodies: YB-1_C-term_ Eurogentec EP085177 (1:200); YB-1_acetyl_ Eurogentec EP085176 (1:200); goat anti-rabbit IgG Fab Fragment FITC, JIR, 111–096–144, (1:250); rabbit IgG-FITC isotype, Southern Biotech, 0111–02 (1:200). Confirmation of antibody specificity and lack of cross-reactivity is described elsewhere^8^.

### Statistics

Data management and statistical analysis was performed using GraphPad Prism software v7.03 (GraphPad Software, San Diego, CA, USA) and SPSS software, v24 (IBM Corp, Armonk, NY, USA). Data were tested for Gaussian distribution using the Kolmogorov-Smirnoff test, confirming non-parametric distribution of MFI YB-1_C-term_, YB-1_acetyl_, and serum levels of galectin, tenascin-C, uPAR, IL-10, IL-1β, NTproANP, M-CSF, ICAM, IL-6, IP10, CCL2, IFNγ, IL-1α, progranulin, midkine, and CCL5. Mann-Whitney U test was used to analyze differences between groups and Sperman’s rho coefficients were calculated to correlate YB-1 expression with cytokines and mortality. Unadjusted significant p-values are highlighted (<0.05*, < 0.005**, < 0.0005***). The probability of survival from the entry in the study (defined as October 2012) to the terminal event of 72 month was estimated by the Kaplan-Meier method.

## Results

Previously we have shown that dialysis patients possess an altered distribution of peripheral blood monocytes presenting fewer classical and more intermediate and non-classical monocytes^8^. The high incidence of bacterial infection in dialysis patients is largely attributed to monocyte dysfunction^25^. This prompted us to compare the reactivity of monocytes between healthy controls (n = 34) and dialysis patients (n = 45). The study cohort consists of 45 patients (14 female, 31 male) that are on dialysis with a mean age of 63 years. The descriptive statistics of the whole dialysis cohort are summarized in Tables 1 and 2.

**Table 1:** Bibliography of dialysis and healthy control cohort.

	dialysis patients n = 45	healthy controls n = 34
age (yr)	63 ± 16 (25–87)	49 ± 6 (40–62)
Gender n (%)	female: n = 14 (31%)	female: n = 13 (38%)
	male: n = 31 (69%)	male: n = 21 (62%)
body weight [kg]	74 ± 15 (50–121)	
time on dialysis (yr)	4.1 + /− 4.0	
dialysis dosage (kt/V)	1.6 + /− 0.7

**Table 2:** Summary of the sample characteristics of the dialysis cohort (n = 45).

Variables	Mean *(Min, Max)*
white blood cells [10³/mm³]	**6.8** *(2.84, 12.84)*
Albumin [g/l]	**38.9** *(32.4, 43)*
Cholesterol [mmol/l]	**4.5** *(2.35, 7.59)*
Triglyceride [mg/dl]	**2.1** *(0.59, 4.7)*
Glucose [mmol/l]	**6.5** *(3.91, 14,17)*
Phosphate [mg/dl]	**1.7** *(0.97, 3.47)*
Calcium [mg/dl]	**2.2** *(1.8, 2.54)*
Vitamin D [µg/l]	**87.6** *(41.7, 163.8)*
Cholesterol [mmol/l]	**4.5** *(0, 8.83)*
HbA1c	**5.9** *(5, 8.5)*
CrP [mg/l]	**0.6** *(0.02, 2.44)*

**Figure 1:**
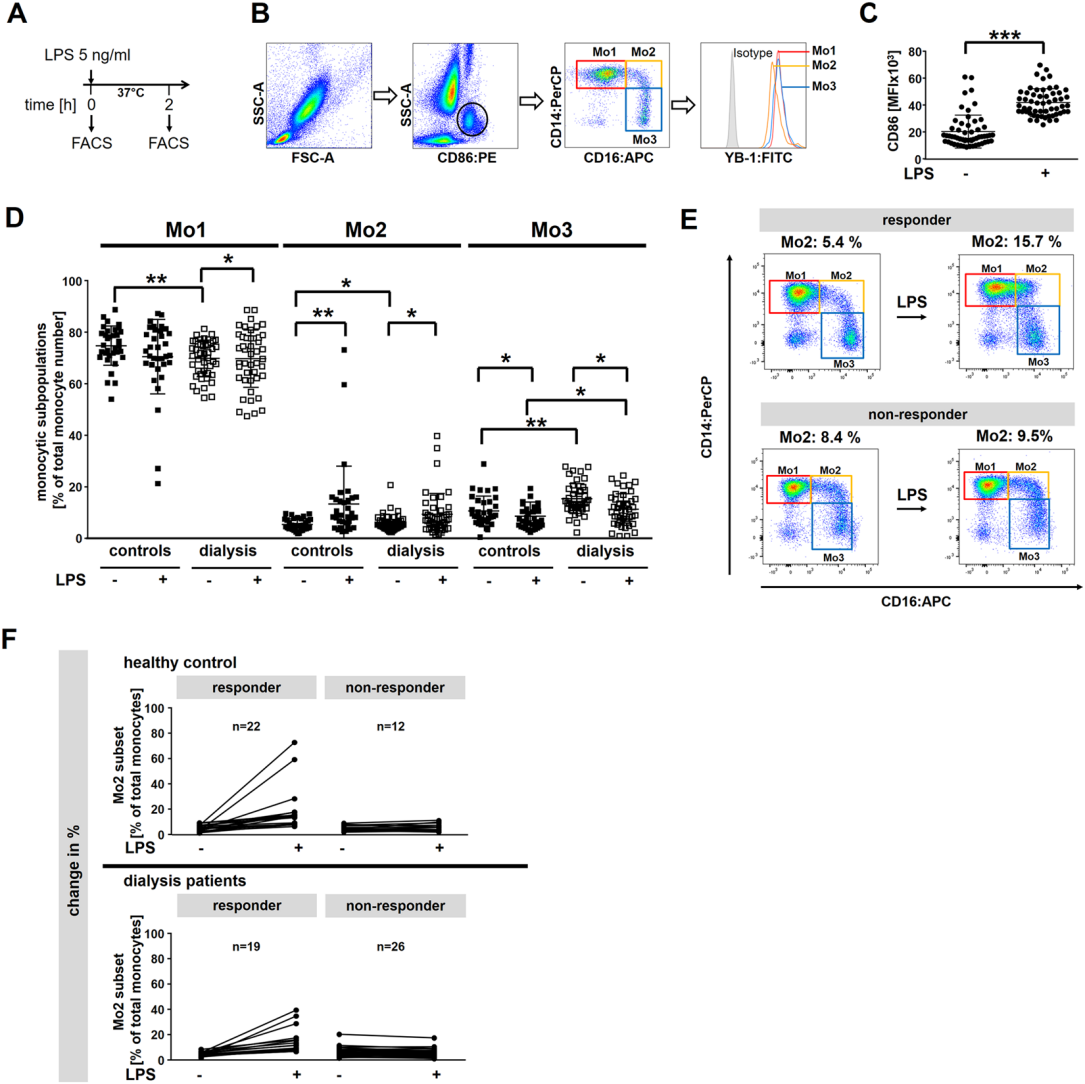
Response of monocytes from healthy controls and hemodialysis patients upon LPS stimulation. **(A)** Experimental design is shown. Whole blood from healthy controls (n = 34) and dialysis patients (n = 45) was incubated with 5 ng/ml LPS for 2 hours or left untreated. **(B)** Cells were analyzed by flow cytometry using the FlowJo Software. Monocytes were gated in a SSC/CD86^+^ dot plot identifying monocytes as CD86^+^ cells with monocyte scatter properties. Monocyte subpopulations (Mo1, Mo2, Mo3) are defined according to their surface expression pattern of the LPS receptor CD14 and the Fcγ receptor CD16. Histogram analysis was used to identify intracellular YB-1_acetyl_ content in the respective monocyte subpopulations. **(C)** Scatter plot illustrating the enhanced CD86 expression on leukocytes following LPS stimulation. **(D)** Scatter plots illustrating the distribution of monocyte sub-populations Mo1-Mo3 following LPS stimulation in dialysis patients *versus* healthy controls. **(E)** Representing examples of CD14/CD16 dotplots defining the responder and non-responder group. **(F)** Diagram representing the change of Mo2 distribution following LPS stimulation in healthy controls and dialysis patients. Positive response was defined with a change of > 10% cells following LPS. Samples/patients with a change of Mo2 cells of less < 10% were classified in the non-responder group. (level of significance: *p < 0.05; **p < 0.005).

**Figure 2:**
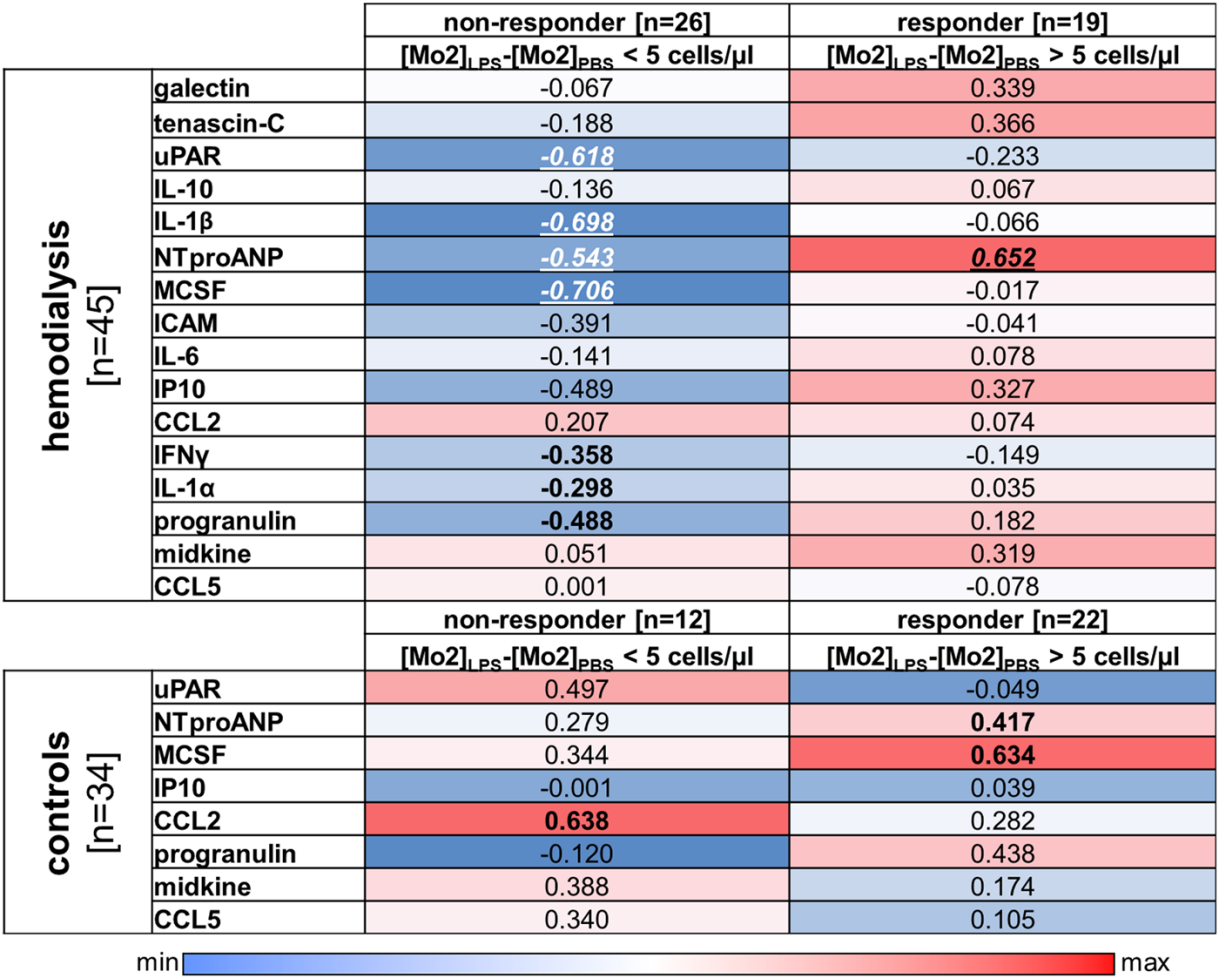
Comparison of responders and non-responders of the hemodialysis and control cohort with systemic cytokine values. Healthy controls and hemodialysis patients were divide into non-responders or responders as described in Fig. 1 and the correlation to systemic cytokine values determined. Data are presented as a heat map, see scale below. Blue represents a negative correlation, whereas red indicates a positive correlation coefficient. Correlation of cytokine/chemokine concentrations was performed by calculating Spearman’s rank correlation coefficients. Levels of significance are provided (Spearman rho analysis; bold p < 0.05; bold and italic: p < 0.005; bold, italic, underlined: p < 0.05 following Bonferroni correction).

To this end, whole blood was drawn and stimulated with LPS (5 ng/ml) for two hours or left untreated (Fig. 1A). Cells were stained for CD86, CD14 and CD16 surface expression to distinguish monocyte subsets (Fig. 1B). Although the expression of CD86, a marker for LPS activity, is enhanced in healthy controls and dialysis patients (Fig. 1C), the responsiveness of monocytes to LPS is differently regulated between healthy controls and dialysis patients. As the number of classical monocytes increases, the number of non-classical monocytes decreases. However, the number of intermediate Mo2 monocytes appears unchanged (Fig. 1D). Monocytes from healthy controls respond in a similar fashion, except for the Mo2 subset, that shows a significant increase in cell numbers (Fig. 1D). Next, by subtracting the number of Mo2 cells prior to LPS from the number following LPS we established the relative responsiveness of monocytes to LPS (Fig. 1E). By doing so we could classify two different subgroups (responders and non-responders) according to their change of Mo2 monocyte numbers following LPS stimulation. Assuming 50 cells/µl of Mo2 monocytes in the blood, we grouped patients/samples with an increase of ≥ 10% as responders and < 10% as non-responders. Applying these criteria there were 40% responders and 60% non-responders in the dialysis cohort. The distribution of responders and non-responders was invers in the control group (Fig. 1F). Since monocytes are a key source of inflammatory cytokines, we extended our analyses and determined the cytokine and chemokine levels in plasma samples collected at the time of cell isolation. In a previous analysis we compared the levels of monocytic YB-1_c-term_ and YB-1_acetyl_ content intracellularly to the cytokine/chemokine content in the plasma^8^. Here we extend our analysis to include the comparison of responders and non-responders with systemic cytokine values **(**Fig. 2). For the non-responders ([Mo2]_LPS_-[Mo2]_PBS_ < 5 cells/µl) in the dialysis cohort, a negative correlation with uPAR, IL-1β, ANP, M-CSF, ICAM, IP10, and progranulin serum levels was observed. In contrast, for responders in the healthy control cohort a positive correlation was determined for M-CSF, progranulin and ANP levels. For the responders ([Mo2]_LPS_−[Mo2]_PBS_ ≥ 5 cells/µl) within the dialysis cohort only the amount of serum ANP positively correlated with ΔMo2 values. In the non-responder group of healthy controls levels of CCL2 positively correlated with Mo2 cell numbers **(**Fig. 2**)**.

**Figure 3:**
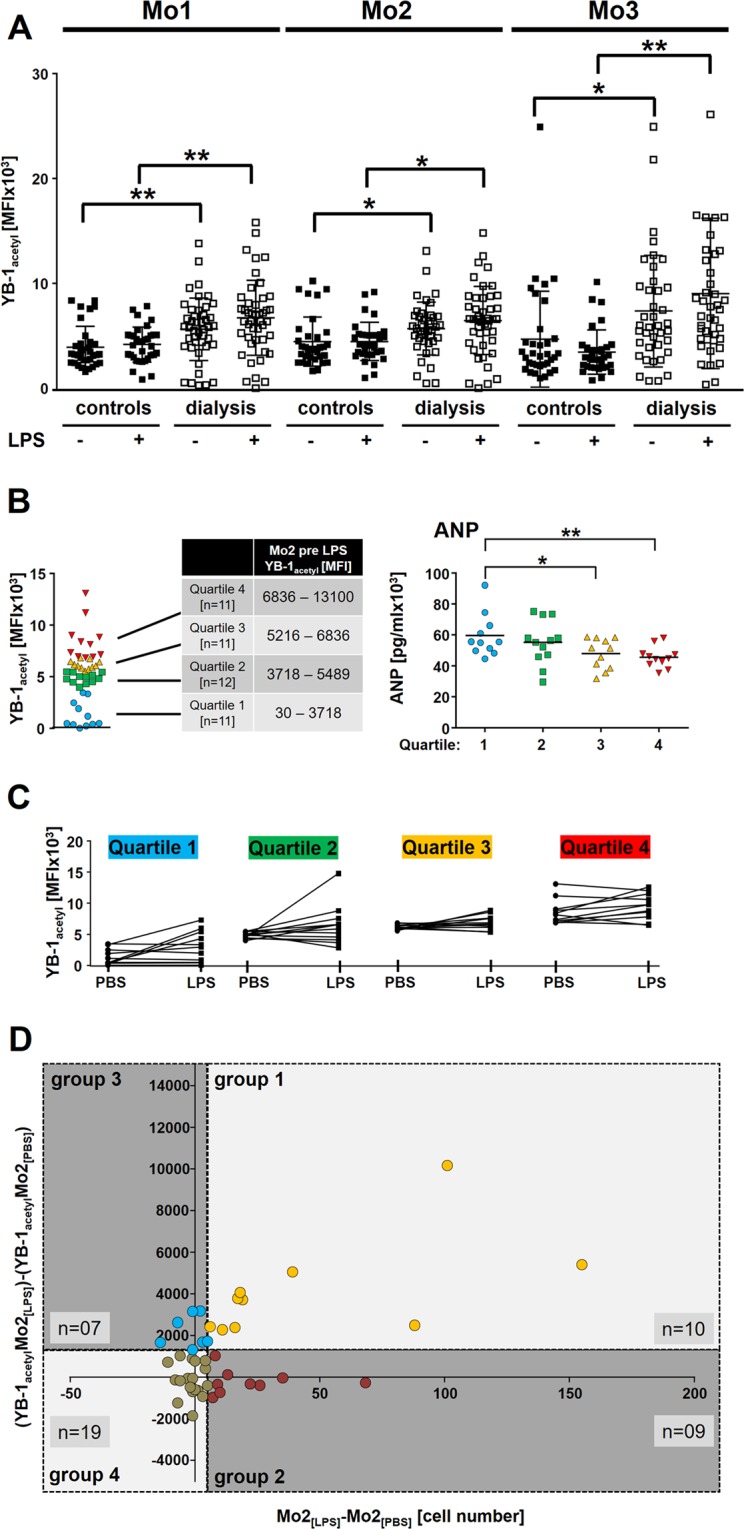
Variability of YB-1_acetyl_ content in monocyte sub-populations following LPS incubation. **(A)** Scatter plots for intracellular YB-1_acetyl_ content in monocyte subpopulations Mo1-Mo3 following LPS stimulation of healthy controls and dialysis patients. **(B)** YB-1_acetyl_ values were grouped into 4 quartiles according to their YB-1_acteyl_ values and analyzed for ANP serum levels. **(C)** Diagram representing the change of intracellular YB-1_acetyl_ in Mo2 sub-population following LPS in dialysis patients according to the quartile 1–4 grouping. **(D)** Dotplot analysis of ΔYB-1_acetyl_ values and ΔMo2 cell numbers following LPS incubation. The threshold values for ΔMo2 were determined as 5 and for ΔYB-1_acetyl_ as 1500. (level of significance: *p < 0.05; **p < 0.005).

LPS is a key component of gram-negative bacteria that binds to specific surface receptors and thereby initiating signaling cascades that lead to cell activation, which includes the activation/phosphorylation of YB-1 within the cold shock domain^11,26^. The phosphorylation of YB-1 induces its nuclear translocation. We have previously shown that acetylated YB-1 is primarily found within the nucleus and observed that protein acetylation is a prerequisite for YB-1 secretion^8,9^. Nuclear YB-1 acts as a transcription factor for several cytokines. We analyzed the intracellular monocytic YB-1_acetyl_ content before and after LPS stimulation to see whether (i) the basal intracellular YB-1_acetyl_ level is altered in dialysis patients compared to heathy controls and (ii) whether it can be used as a marker of cell responsiveness to LPS. Monocytes from dialysis patients show a significantly higher content of acetylated YB-1 compared to healthy controls (Fig. 3A), which likely reflects the “pre-activated” cell phenotype. However, YB-1_acetyl_ levels did not significantly change following LPS challenge, when analyzed in the whole dialysis cohort (n = 45). In contrast, Mo2 monocytes from healthy controls exhibited an increase of intracellular YB-1_acetyl_ content. Given the high variability of YB-1_acetyl_ levels in Mo2 we divided the responsiveness within the dialysis cohort into 4 quartiles based upon their basal YB-1_acetyl_ levels (Fig. 3B). Analysis of the corresponding YB-1_acetyl_ values shows that patients with the lowest YB-1_acetyl_ content have a higher probability to react with an increase of YB-1_acetyl_ after LPS stimulation (Fig. 3C). Patients in the 1^st^ quartile with the lowest YB-1_acetyl_ content have significantly higher levels of ANP compared to patients within the 3^rd^ and 4^th^ quartiles for YB-1_acetyl_ content.

**Figure 4:**
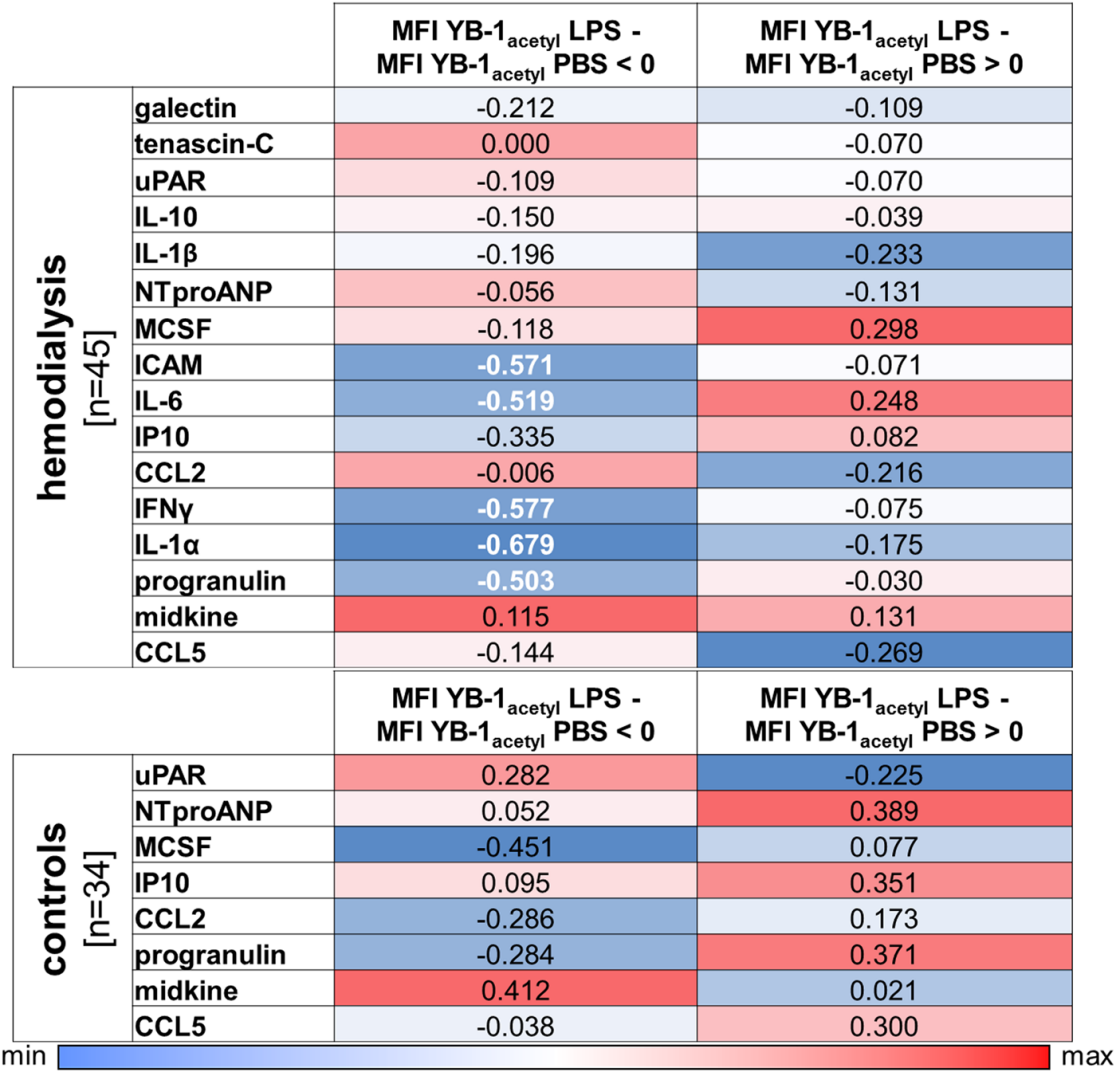
Comparison of ΔYB-1_acteyl_ values of the hemodialysis and control cohort with systemic cytokine values. Healthy controls and hemodialysis patients were divided into non-responders or responders as described in Fig. 3 and the correlation to systemic cytokine values determined. Data are presented as a heat map, see scale below. Blue represents a negative correlation, whereas red indicates a positive correlation coefficient. Correlation of cytokine/chemokine concentrations was performed by calculating Spearman’s rank correlation coefficients. Levels of significance are provided (Spearman rho analysis; bold p < 0.05).

Next we analyzed the correlation between LPS responsiveness, determined by ΔMo2 cell numbers, and cold shock protein acetylation status (YB-1_acetyl_ with LPS - YB-1_acetyl_ with PBS). Most of the dialysis patients exhibit fewer Mo2 cells and even less YB-1_acetyl_ content following LPS stimulation, indicating that the monocytes of dialysis patients are already “pre-activated” and do not respond in a “normal fashion” (Fig. 3D). Another point supporting the notion of the “pre-activated” monocyte hypothesis is that several cytokines are elevated in dialysis plasma samples compared to healthy controls^8^. YB-1 is known to regulate the expression of a number of cytokines and non-classical monocytes are potent producers of pro-inflammatory cytokines^27,28^. Therefore we aimed to correlate intracellular monocytic YB-1_acetyl_ levels in response to LPS with cytokines/chemokine serum levels (Fig. 3C). To do so, we calculated the ΔMFI of YB-1_acetyl_ in response to LPS: ΔMFI = YB-1_acetyl_ Mo2_LPS_ - YB-1_acetyl_ Mo2_PBS_ and grouped the patients regarding ΔMFI below or above 0 (Fig. 3D). In the dialysis group with ΔMFI YB-1_acetyl_ below 0 a negative correlation to ICAM, IL-6, IFNγ, IL-1α, and progranulin is seen. That was not the case in the group with ΔMFI YB-1_acetyl_ above 0. All the other cytokines are not interrelated in calculations with both groups (Fig. 4). In the healthy control group, a negative correlation of ΔMFI YB-1_acetyl_ below 0 to M-CSF within the serum was determined (Fig. 4).

In the dialysis cohort, no correlation could be found using the YB-1_acetyl_ levels prior to LPS stimulation indicating that the responsiveness of the monocytes to LPS is decisive and not the YB-1_acetyl_ contents *per se*.

**Figure 5:**
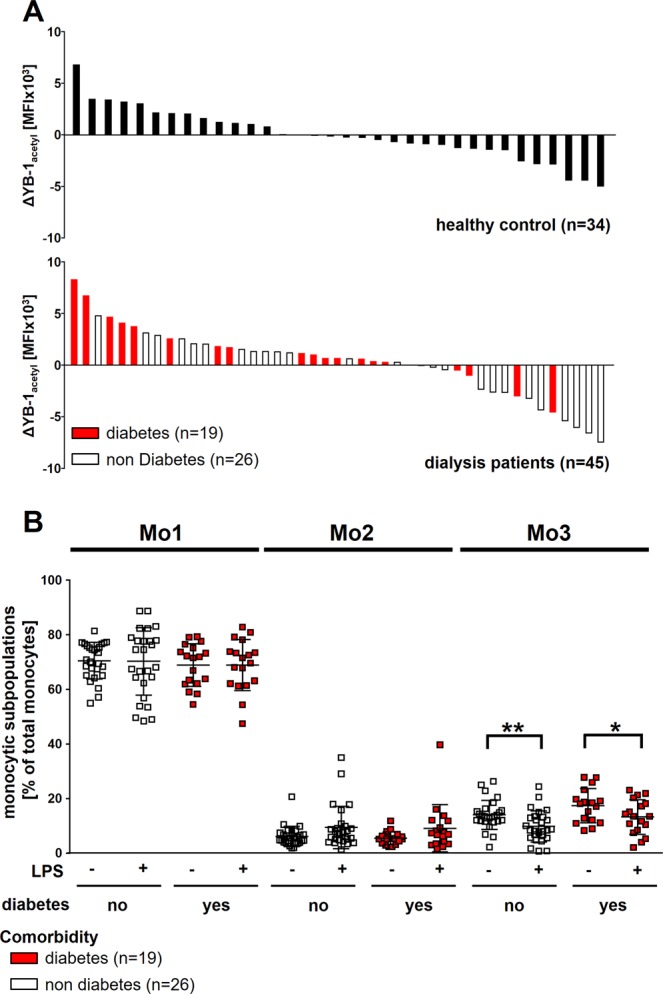
Comorbidities and intracellular YB-1_acetyl_ content. **(A)** Diagram showing the distribution of YB-1_acetyl_ following LPS in dialysis patients and healthy controls. Most dialysis patients suffering from diabetes (red bar graphs) react with an increase of intracellular YB-1_acetyl_ following LPS incubation. **(B)** Scatter plots showing the distribution of monocyte sub-populations Mo1-Mo3 following LPS incubation in dialysis patients without and with diabetes. (level of significance: *p < 0.05; **p < 0.005).

Remarkably, monocytes from dialysis patients show a wide spectrum of acetylated YB-1 levels, which becomes even broader after LPS challenge. This provokes the question as to whether comorbidities might be correlated to the different monocyte responses. Assigning monocyte YB-1_acetyl_ levels obtained after LPS stimulation to individual patients, we find that 14 of the 28 patients with high YB-1_acetyl_ content in monocytes suffer from diabetes mellitus, compared to only 5 of 19 patients diagnosed with diabetes showing lower YB-1_acetyl_ content in monocytes after LPS challenge (Fig. 5A, diabetes patients are highlighted in red). To see whether the rise in acetylated YB-1 upon LPS stimulation in diabetes patients can be attributed to a distinct monocyte subpopulation, we first determined the monocyte distribution. Diabetes and non-diabetes patients showed a comparable rise in Mo1, no change in Mo2, and decreased number of Mo3 monocytes following LPS stimulation (Fig. 5B). Although the YB-1_acetyl_ levels within distinct monocyte subsets are unchanged, monocytes from diabetes patients mostly react with an increase in YB-1_acetyl_ in monocytes following LPS challenge in contrast to non-diabetes patients.

### Are serum cytokine levels indicative of patient survival?

Although the cohort of patients within study was relatively small, we analyzed the 6-year survival data to get a first insight into whether the cytokine levels correlate with patient survival. Among many other causes, chronic inflammation is one of the main reasons for the increased morbidity and mortality in hemodialysis patients^3,29^. In our dialysis cohort more than 50% of the patients died within 6 years (Supplementary Fig. 2A). We compared the baseline values of the cytokines mentioned above (Fig. 4) as well as the MFI of YB-1_acetyl_ determined in Mo2-monocytes between the survival and mortality groups 6 years post analyses. Supplementary Fig. 2B shows that the MFI of YB-1_acetyl_ in the group of deceased patients was higher compare to the values in the surviving group. On the basis of the receiver-operator curve (ROC), the most appropriate cut-off point for MFI of YB-1_acetyl_ to predict mortality was 6444 (sensitivity 41%, specificity 94%, area under the curve (AUC) 0.750, Supplementary Fig. 2C,D). The risk to die, in a time frame of 6 years, is significantly enhanced if the MFI of YB-1_acetyl_ was higher than 6444. In addition the deceased patients exhibited higher titers of IL-6 (14.3 pg/ml *versus* 6.5 pg/ml), uPAR (1457 pg/ml *versus* 1020 pg/ml), and IP10 (78.5 pg/ml *versus* 52.2 pg/ml) (Supplementary Fig. 2E,F). Subsequently, ROC curve analyses were carried out for the mentioned cytokines alone or in combination (Supplementary Table 1**)**. A combined analysis of several cytokines or with YB-1_acetyl_ shows an overall improvement of the ROC curves. This is most evident when uPAR and YB-1_acetyl_ are combined (AUC reaching 0.803).

## Discussion

Monocytes circulate within the blood looking for “danger signals” and are among the first cells to enter sites of inflammation. Monocytes phagocytose and present antigens, secrete chemokines, and proliferate in response to activation. During the last years, numerous metabolic factors like glucose, lipoproteins as well as various pro- and anti-inflammatory mediators have been identified that induce monocyte activation within the circulation^21,30^. Functional studies on monocytes from dialysis patients identified an increased production of pro-inflammatory cytokines and reduced capacity for antigen presentation^31^. In this pilot study, we have identified the YB-1 acetylation status as an indicator of monocyte responsiveness. We also find enhanced systemic levels of pro-inflammatory cytokines (IL-6, uPAR, and IP-10) and show that monocytes from dialysis patients are”pre-activated”, as indicated by their reduced responsiveness to inflammatory stimuli like LPS.

The novelty of our study is the investigation of monocyte responsiveness in whole blood to LPS stimulation. We focused on the Mo2 population as this is where we observed changes in absolute number and posttranslational modifications of YB-1 following LPS. One task was to define responders and non-responders, based on an increase or decrease in monocyte numbers. To normalize the responsiveness we calculated the relative increase of Mo2 cells. The arbitrary threshold between the two groups was chosen as a 10% increase in the number of Mo2 cells indicating a positive response. This definition resulted in 40% positive responders in the dialysis cohort (19 of 45 patients) versus 64% (22 of 34 people) of the control cohort. It is striking that for the majority of dialysis patients, monocytes no longer react to LPS. One hypothesis is that the LPS is no longer able to activate intracellular signaling cascades in these cells; although CD86 expression is enhanced following LPS. This idea is supported by studies which describe monocytes from dialysis patients as “pre-activated”^15,25,32^.

One should keep in mind that unlike healthy individuals, the monocytes of hemodialysis patients are under uremic basal conditions and are exposed to artificial materials 3 times per week; the latter consisting of plastic tubing and polymer filters. During a 4 hour dialysis session, each cell passes through the filter approximately 10 times. Mechano-stimulation by sheer force or pressure may activate cells^8,25^.

Experimental data of our group demonstrate that Y-box binding protein-1 (YB-1) is up-regulated during monocyte activation^8^. Patients with high YB-1_acetyl_ content show enhanced levels of pro-inflammatory cytokines within the serum, which is indicative of a chronic state of activation that results in exhaustion within the monocyte population^20^. Thus, monocyte exhaustion represents a state of immune paralysis that predisposes the affected individuals to infection, thereby raising the rate of morbidity^20^. Particularly amongst patients with diabetes mellitus, our data indicate that monocyte exhaustion appears to be predictive of an increased rate of mortality. Considering that this prediction is based upon a single measurement, the chosen parameters appear to indicate the existence of a predetermined course of events leading to death, which we call the “legacy effect”. Indeed a similar observation was made in a murine sepsis model, where plasma IL-6 levels 6 hours post infection predicted life or death^33^. Several years ago Kato *et al*. identified elevated monocyte numbers as independent predictor of total and cardiovascular mortality in hemodialysis patients^34^.

Monocyte differentiation is regulated by numerous transcription factors including NF-κB, FOS, KLF2, TFEC, and RUNX to name but a few, which endow each subset with unique gene signatures and metabolic profiles^35^. YB-1 is known to regulate the expression of a number of oxidative phosphorylation proteins as well as cytokines^27,36^. Recently, we described that the acetylation status of YB-1 is higher in circulating monocytes of dialysis patients compared to healthy controls, which correlated with differential cytokine content in serum^8^. Within this study we extend our analysis by identifying responders and non-responders with regard to cytokine production (Fig. 2). Taking advantage of the long follow up and associated deaths, we correlate the risk to die with the cytokine profile and YB-1_acetyl_ content. By doing so we found a positive correlation of acetylated YB-1, IL-6, uPAR, and IP-10 with mortality.

A number of studies have shown that hemodialysis and renal transplant patients have elevated IL-6 serum levels^20,37^. IL-6 has a very marked pleiotropy, being involved not only in inflammation, but also in the regulation of several fundamental organ functions by promoting myocardial fibroblast proliferation, stimulating thermogenesis, suppressing the thyroid axis, and inducing hormone secretion^37^. However, the causes of elevated IL-6 concentration in dialysis patients could be due to decreased renal elimination and/or increased generation following induction by various factors such as uremic toxins, oxidative stress, volume overload, co-morbidities, and other dialysis-related factors- such as artificial membranes, nonsterile dialysate and back filtration^38,39^. The pluripotency of effects makes IL-6 unique among the pro-inflammatory cytokines and indicates that it represents not only a potential agent of organ damage in pathophysiologic conditions, but also a potent marker of the overall severity of the inflammation process.

Our findings that IL-6 has a predictive power (AUC = 0.675; sensitivity of 26.6% and specificity of = 88.9%) for determining mortality in dialysis patients is in accordance with findings that described elevated IL-6 levels in peritoneal dialysis patients^40^. Furthermore, IL-6 has recently been evaluated as a biomarker to diagnose sepsis using a biosensor to detect elevated IL-6 levels in blood samples^20,41^ and IL-6 predicts mortality in murine models of sepsis^33^. Soluble urokinase plasminogen activator receptor (uPAR) is present at low concentrations in healthy individuals, while higher levels have been observed in persons with infection, inflammation, as well as in dialysis patients^42–44^. Several studies described an association of high uPAR levels with mortality in dialysis patients^42,45^^,^. Wlazel *et al*. described that elevated uPAR levels provide independent information on all-cause mortality risk in patients undergoing dialysis^42^. Within our study we showed that the combined use of uPAR and YB-1_acetyl_ provides a higher predictive power for death in comparison to uPAR alone.

However, our study has its limitations. First and foremost being the relatively small size of our patient cohort. Secondly, although YB-1 is known to regulate the expression of a number of genes, data conclusively linking specific activities with the YB-1 acetylation status are lacking. Since acetylated YB-1 is known to be secreted, the possibility that paracrine or endocrine activities of extracellular YB-1 exist that might influence survival cannot be excluded.

To our knowledge, this is the first study linking post-translational modification of YB-1 to an increased risk of death in patients. Previously, we have demonstrated that the polarization of murine macrophages is dependent on YB-1 expression^26^. Additionally, we showed that the differential regulation of CCL5 gene expression during monocyte/macrophage differentiation is dependent upon the phosphorylation status of YB-1^10,12^. Before proceeding to suggest that YB-1_acetyl_ content be used to monitor a patient’s immune status, additionally studies demonstrating a causal relationship between YB-1_acetyl_ content and cytokine secretion are required. Once such data exist, it would then be of interest to test whether specific intervention strategies with acetyl transferase inhibitors (*e.g*. curcumin) can restore functionality to exhausted monocytes *ex vivo*. One must keep in mind that such interventions may alter the epigenetic status of the cell. Such approaches are already being investigated for cancer; a condition where immune cell exhaustion in T lymphocytes has already been identified as an underlying cause. It would therefore be of interest to determine the YB-1_acetyl_ content within these T cells to see whether it regulates the expression of inhibitory receptors like CTLA4 and PD-1, which enforce the unresponsive state of these cells. PD-1 has also recently been described as a marker of monocyte exhaustion^46,47^. Since cancerous cells utilize an altered metabolism, known as the Warburg effect, it would also be of interest to determine to what extent this contributes to changes in lysine acetylation, which utilizes acetyl-CoA as a substrate, and whether altering a cells metabolism may help to restore functionality.

## Supplementary information

Supplementary Data
