# Supplementary material for: Altered monocytic phenotypes are linked with systemic inflammation and may be linked to mortality in dialysis patients
Source: Sci Rep. 2019 Dec 13;9:19103. doi: 10.1038/s41598-019-55592-y (PMC6911068; doi:10.1038/s41598-019-55592-y)
Supplement: Supplementary file 1 — Supplementary Data [file 41598_2019_55592_MOESM1_ESM.pdf]

Altered monocytic phenotypes are linked with systemic inflammation and may be linked to mortality in dialysis patients

Sabine Brandt, Lara Ewert, Florian G. Scurt, Charlotte Reichardt, Jonathan A. Lindquist, Xenia Gorny, Berend Isermann[1], Peter R. Mertens[#]

Department of Nephrology and Hypertension, Diabetes and Endocrinology, Otto-von-Guericke University Magdeburg, Magdeburg, Germany

[1]Institute of Clinical Chemistry and Pathobiochemistry, Otto-von-Guericke University Magdeburg, Magdeburg, Germany

Supplementary Figure 1

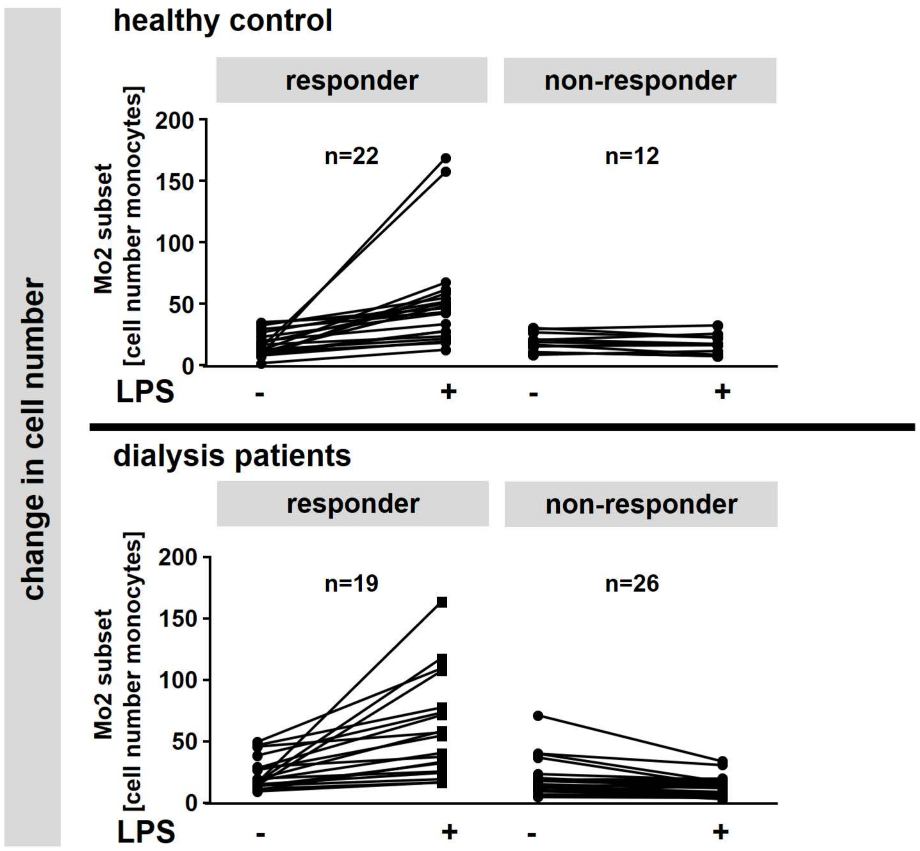

Supplementary Figure 1. Diagram representing the changes of Mo2 cell numbers following LPS stimulation in healthy controls and dialysis patients.

Supplementary Figure 2

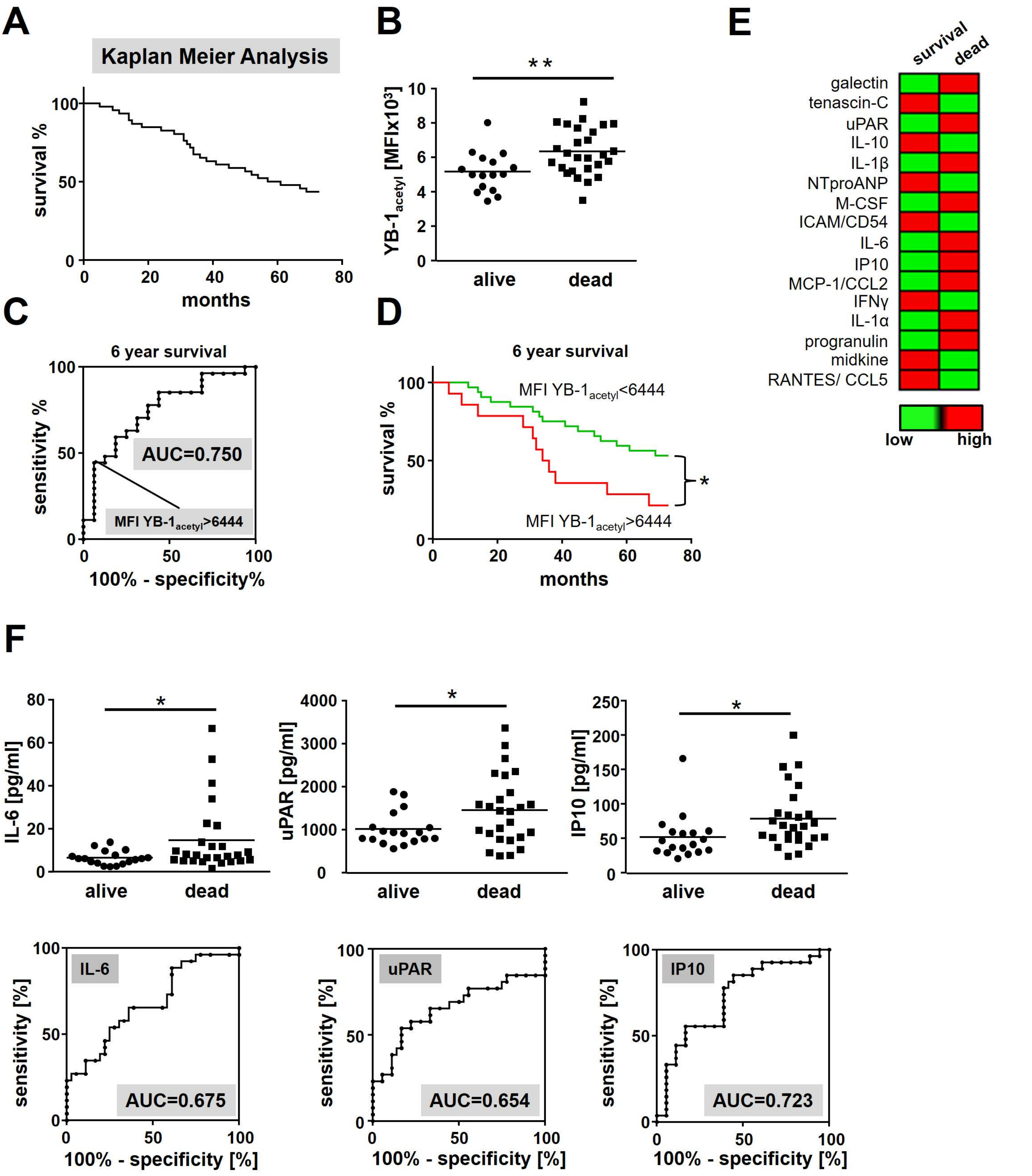

Supplementary Figure 2. Correlation of intracellular YB-1acetyl levels and serum cytokine values with survival/mortality of dialysis patients. (A) Kaplan-Meier curve of all dialysis patients within this study. (B) Diagram showing the intracellular YB-1acetyl values of alive and dead patients. (C) Receiver-operator-curve analysis of the YB-1acetyl levels of alive and dead patients. (D) Kaplan-Meier curves by MFI of YB-1acetyl above or less 6444. (E) Heat map analysis of cytokines/chemokines comparing the survival and dead group of dialysis patients. (F) Diagrams illustrating the serum levels of IL-6, uPAR, and IP10 of the survival group compared to the deceased patients. Below the corresponding receiver-operater-curve analysis is shown. (level of significance: *p<0.05; **p<0.005)

Supplementary Table 1

ROC curve analyses for the mentioned cytokines alone or in combination.

	IL6	uPAR	IP10	YB-1$_{acetyl}$
**AUC for one parameter alone**	0.675	0.654	0.723	0.750
**AUC combined with YB-1$_{acetyl}$**	0.791	**0.803**	0.796	

	IL6	uPAR	IP10	YB-1$_{acetyl}$
**IL6**		0.747	0.765	0.769
**uPAR**	0.747		0.703	**0.803**
**IP10**	0.765	0.703		0.769
**YB-1$_{acetyl}$**	0.769	**0.803**	0.796	
